# Molecular detection and genetic characterization of infectious laryngotracheitis virus in poultry in Myanmar

**DOI:** 10.1186/s12917-020-02666-z

**Published:** 2020-11-23

**Authors:** Zhiyuan Yang, Shiro Murata, Sotaro Fujisawa, Masaki Takehara, Ken Katakura, Myint Myint Hmoon, Shwe Yee Win, Saw Bawm, Satoru Konnai, Kazuhiko Ohashi

**Affiliations:** 1 Faculty of Veterinary Medicine, Hokkaido University, Sapporo, Japan; 2 Institute of Animal Husbandry and Veterinary Medicine, Beijing Academy of Agriculture and Forestry Sciences, Beijing, China; 3 University of Veterinary Science, Yezin, Nay Pyi Taw, Myanmar

**Keywords:** Infectious laryngotracheitis virus, Molecular detection, Myanmar, Poultry, Phylogeny

## Abstract

**Background:**

Avian infectious laryngotracheitis (ILT) is a highly contagious viral disease that causes severe economic losses to the poultry industry worldwide. In Southeast Asian countries, including Myanmar, poultry farming is a major industry. Although it is known that infectious respiratory pathogens, including infectious laryngotracheitis virus (ILTV), are a major threat to poultry farms, there are no data currently available on the epidemiology of ILTV in Myanmar. Therefore, in this study, we conducted a molecular detection of ILTV in 20 poultry farms in Myanmar.

**Results:**

Of the 57 tested oropharyngeal swabs, 10 were positive for ILTV by polymerase chain reaction of a 647 bp region of the thymidine kinase (TK) gene, giving a prevalence of ILTV of 17.5% (10/57). Further sequencing analysis of infected cell protein 4 (*ICP4*) gene and glycoprotein B, G, and J (*gB*, *gG*, and *gJ*) genes indicated that these isolates were field strains. Phylogenetic analysis revealed that the Myanmar strains clustered together in a single branch and were closely related to other reference strains isolated from Asian countries.

**Conclusions:**

This study demonstrated the presence of ILTV in poultry farms in Myanmar. The genetic characterization analysis performed provides the fundamental data for epidemiological studies that monitor circulating strains of ILTV in Myanmar.

**Supplementary Information:**

**Supplementary information** accompanies this paper at 10.1186/s12917-020-02666-z.

## Background

Infectious laryngotracheitis (ILT) is an acute and highly contagious viral disease that affects adult chickens, which is characterized by inflammation and hemorrhage of the larynx and trachea [1]. The etiological agent is *Gallid alphaherpesvirus 1* (GaHV-1), a member of the family *Herpesviridae*, subfamily *Alphaherpesvirinae*, genus *Iltovirus*, and is commonly called infectious laryngotracheitis virus (ILTV) [2]. Acute ILTV infection can cause high morbidity and mortality in chickens while chronic infection is characterized by decreased growth rates and reduced egg production [3], thus causing serious economic losses to the poultry industry worldwide.

In Southeast Asian countries, including Myanmar, poultry farming is a major industry. Myanmar, which is located in the northeast edge of Southeast Asia, is the largest country in the mainland of Southeast Asia. Along with the increasing demand for sustainable, locally produced, and safe poultry products for Myanmar consumers, the prevention and control of infectious diseases in poultry has become increasingly important.

Currently, immunization is the principal tool used to control ILTV [4]. The two types of live-attenuated vaccines used are derived from either chicken embryo-origin (CEO) vaccine strains [5], which are attenuated by serial-passage in embryonated eggs, or tissue culture-origin (TCO) vaccine strains [6], which are obtained by multiple passages in tissue culture. However, the vaccine strains can revert to virulence after passages in susceptible birds [7]. Menendez et al indicated that live-attenuated vaccine-related isolates may have contributed to ILT outbreaks worldwide [8]. ILT still occurs frequently and causes significant economic losses in the chicken industry of many countries including Italy [9], Korea [10], Australia [11], and China [12], despite the preventative and biosecurity measures that are in place. It is reported that some ILTV isolates involved in recent ILT outbreaks in Italy might have originated from CEO vaccines [13]. Meanwhile, virulent field strains genetically related to CEO vaccines have also been detected in Korea [10]. Another reason leading to that might be related with the recombination events between attenuated ILTV vaccines resulting in more virulent or transmissible field strains [14]. Two newly emerged genotypes of viruses have been proven to be a result of the recombination between a previously existing Australian vaccine strain (SA2 and A20) and a vaccine (Serva) introduced into the country in 2007 [14, 15]. Therefore, investigating ILTV strains in circulation in endemic areas is not only useful to evaluate vaccine efficacy, but also necessary to identify the etiology of disease outbreaks in the poultry population.

Since some ILTV field strains are closely related to the vaccine-derived strains, most studies have used polymerase chain reaction–restriction fragment length polymorphism (PCR-RFLP) or sequencing a single target region to discriminate between them [9]. However, sequencing multiple target regions would be more useful to better characterize circulating strains and enable more reliable discrimination between ILTV field and vaccine-derived strains [16].

To date, the avian influenza and Newcastle disease have been reported in Myanmar [17–19]. More recently, the genetic characteristics of other respiratory pathogens, including *Mycoplasma gallisepticum*, *Mycoplasma synoviae*, and infectious bronchitis virus, have also been investigated [20]. However, to date, there is no scientific report on the epidemiology of ILTV in Myanmar, although some clinical signs suggestive of respiratory pathogen infections have been observed.

In this study, therefore, we aimed to assess the prevalence of ILTV in chickens from major Myanmar poultry farms using molecular detection techniques, and perform Sanger sequencing of the isolates to monitor the strains in circulation in this region.

## Results

### Molecular detection of ILTV in poultry farms of Myanmar

Chicken swab samples (*n* = 171) were collected from 20 poultry farms in Myanmar; three samples were pooled and a total of 57 pools were subjected to the DNA extraction and PCR targeting the thymidine kinase (*TK*) gene. Out of 57 pools collected from different farms, 10 (17.5%) were positive for ILTV (Table 1). The DNA positive control extracted from the ILTV attenuated vaccine showed amplification with a band at the expected size (647 bp) after gel electrophoresis. The nucleotide sequences of the amplified target region were further confirmed by sequencing, and no difference in the sequences between the Myanmar samples and vaccine strains, TCO and CEO were observed (Supplemental Table 1). Of note, most of the positive samples had been collected from the Yangon area during the wet season (May) (Table 1).

**Table 1:** Details of the distribution of ILTV

Sampling area	Farm ID	Date	No. of chickens	No. of detected/No. of tested ^a^ (%)
Mandalay	Ma-1	Feb. 10, 2018	12	0/4 (0.0)
	Ma-2	Feb. 10, 2018	9	1/3 (33.3)
	Ma-3	Feb. 10, 2018	9	0/3 (0.0)
	Ma-4	Feb. 11, 2018	9	0/3 (0.0)
	Ma-5	Feb. 11, 2018	9	0/3 (0.0)
Pyin Oo Lwin	Py-1	Feb. 12, 2018	9	0/3 (0.0)
	Py-2	Feb. 12, 2018	9	0/3 (0.0)
	Py-3	Feb. 12, 2018	9	0/3 (0.0)
	Py-4	Feb. 12, 2018	9	0/3 (0.0)
	Py-5	Feb. 12, 2018	9	0/3 (0.0)
Yangon	Ya-1	May 28, 2018	9	2/3 (66.7)
	Ya-2	May 28, 2018	6	2/2 (100)
	Ya-3	May 28, 2018	6	0/2 (0.0)
	Ya-4	May 28, 2018	6	1/2 (50.0)
	Ya-5	May 29, 2018	9	3/3 (100)
	Ya-6	May 29, 2018	6	0/2 (0.0)
	Ya-7	May 29, 2018	9	0/3 (0.0)
	Ya-8	May 29, 2018	9	1/3 (33.3)
	Ya-9	May 29, 2018	9	0/3 (0.0)
	Ya-10	May 29, 2018	9	0/3 (0.0)
Total				10/57 (17.5)

^a^Three oropharyngeal swab samples were pooled and analyzed

### Characterization of the *ICP4*, *gB*, *gG*, and *gJ* genes

To genetically characterize the ILTV isolates, the *ICP4*, *gB*, *gG*, and *gJ* genes were partially amplified in the 10 field samples that were positive for the TK gene. Six samples from different farms (Farm Ma-2, Farm Ya-1, Farm Ya-2, Farm Ya-4, Farm Ya-5, and Farm Ya-8) were selected for sequence analysis; five of the six field samples showed 100% identity with each other although some single nucleotide polymorphisms (SNPs) were also observed in *ICP4*, *gB*, *gG*, and *gJ* genes when compared to reference sequences from GenBank (Tables 2 and 3). For the *ICP4* gene, two fragments located at positions 181–868 and 3645–4268 were used to differentiate ILTV field isolates from live-attenuated vaccine strains as described previously [21]. As shown in Table 2, a 12-bp deletion, two substitutions in the *ICP4* gene fragment 1, and five point mutations in the *ICP4* gene fragment 2 were observed.

**Table 2:** Nucleotide sequence alignment of ICP4 gene fragments from the isolates in Myanmar, vaccine strains and other ILTV strains

Name of strains	Nucleotide position from ATG ^a^													
	ICP4 fragment 1 (positions 181 to 868)								ICP4 fragment 2 (positions 3645 to 4268)					
	259–270	438	456	594	597	611	795	811	3879	3905	3957	3981	4012	4047
Farm Ya-1	* ^b^	A	A	C	A	*	G	G	A	T	C	C	A	A
Farm Ya-2	*	-^c^	–	–	–	*	–	–	–	–	–	–	–	–
Farm Ya-4	*	–	–	–	–	*	–	–	–	–	–	–	–	–
Farm Ya-5	*	–	–	–	–	*	–	–	–	–	–	–	–	–
Farm Ya-8	*	–	–	–	–	*	–	–	–	–	–	–	–	–
Farm Ma-2	*	–	–	–	–	*	–	–	–	–	–	–	–	–
MF417811_USA/14.939	*	–	–	–	–	*	–	–	–	–	–	–	–	–
JN542533_USA/1874C5	GCGGCCCAAGAC	G	G	*	G	G	A	A	–	C	T	–	–	G
JN542534_USA/USDA	GCGGCCCAAGAC	G	–	–	G	*	–	–	–	C	T	T	G	G
JN542535_USA/81658	GCGGCCCAAGAC	G	–	–	G	*	–	–	–	C	T	T	G	G
JN542536_USA/63140	*	–	–	–	–	*	–	–	–	–	–	–	–	–
JN804827_Australia/CL9	*	–	–	–	–	*	–	–	–	–	–	–	–	–
JX646898_Australia/V1–99	*	–	–	–	–	*	–	–	–	C	–	–	–	G
JN596963_Australia/A20 vaccine	GCGGCCCAAGAC	G	G	–	G	*	A	A	–	C	T	–	–	G
HQ630064_Australia/Serva vaccine	*	–	–	–	–	*	–	–	–	–	–	–	–	–
JX458822_China/LJS09	*	–	–	–	–	*	–	–	–	–	–	–	–	–
JX458823_China/WG	*	–	–	–	–	*	–	–	T	–	–	–	–	–
JX458824_China/K317 vaccine	*	–	–	–	–	*	–	–	T	–	–	–	–	–
MH937564_Korea	*	–	–	–	–	*	–	–	–	–	–	–	–	–
MH937565_Korea	*	G	G	–	G	*	A	A	–	–	–	–	–	–
MH937566_Korea	*	–	–	–	–	*	–	–	–	–	–	–	–	–
JN580312/TCO vaccine-IVAX	GCGGCCCAAGAC	G	–	–	G	*	–	–	–	C	T	T	G	G
JN580313/CEO vaccine-TRVX	*	–	–	–	–	*	–	–	–	–	–	–	–	–
NC006623_USA	GCGGCCCAAGAC	G	G	*	G	G	A	A	–	C	T	–	–	G

^a^The ICP4 gene sequence with Genbank accession number NC_006623 was taken as a reference

^b^* Deletions within the sequences

^c^-Regions where the sequences are identical to those of Farm Ya-1

**Table 3:** Nucleotide sequence alignment of gB, gG and gJ gene fragments from the isolates in Myanmar, vaccines strains and other ILTV strains

Name of strains	Nucleotide position from ATG^a^												
	gB	gG					gJ						
	1931	66	102	173	292	344	461	484	765	777	832	878	894
Farm Ya-1	C	G	C	T	C	T	A	C	C	C	A	T	G
Farm Ya-2	-^b^	–	–	–	–	–	–	–	–	–	–	–	–
Farm Ya-4	–	–	–	–	–	–	–	–	–	–	–	–	–
Farm Ya-5	T	–	G	–	–	–	–	–	–	T	–	–	–
Farm Ya-8	–	–	–	–	–	–	–	–	–	–	–	–	–
Farm Ma-2	–	–	–	–	–	–	–	–	–	–	–	–	–
MF417811_USA/14.939	–	A	G	–	–	–	–	–	–	T	–	–	–
JN542533_USA/1874C5	–	–	G	–	A	–	–	–	T	T	–	–	–
JN542534_USA/USDA	–	A	G	–	–	–	–	T	–	T	–	C	–
JN542535_USA/81658	T	A	G	–	–	–	–	T	–	T	–	C	–
JN542536_USA/63140	–	A	G	–	–	–	–	–	–	T	–	–	–
JN804827_Australia/CL9	–	A	G	–	–	–	–	–	–	T	–	–	–
JX646898_Australia/V1–99	–	–	G	G	A	G	–	–	T	T	–	–	–
JN596963_Australia/A20 vaccine	–	–	G	G	A	G	–	–	T	T	–	–	–
HQ630064_Australia/Serva vaccine	T	A	G	–	–	–	–	–	–	T	–	–	–
JX458822_China/LJS09	T	A	G	–	–	–	–	–	–	T	–	–	–
JX458823_China/WG	–	–	G	G	A	G	–	–	T	T	–	–	–
JX458824_China/K317 vaccine	T	A	G	–	–	–	–	–	–	–	–	–	–
MH937564_Korea	T	A	G	–	–	–	–	–	–	T	–	–	–
MH937565_Korea	–	–	G	G	A	G	–	–	T	T	–	–	–
MH937566_Korea	T	A	G	–	–	–	–	–	–	–	–	–	–
JN580312/TCO vaccine-IVAX	T	A	G	–	–	–	–	T	–	T	–	C	–
JN580313/CEO vaccine-TRVX	T	A	G	–	–	–	–	–	–	–	–	–	–
NC006623_USA	T	A	G	–	–	–	–	T	–	T	–	C	–

^a^The sequence of each gene (gB, gG and gJ genes) with Genbank accession number NC_006623 was taken as a reference

^b^-Regions where the sequences are identical to those of Farm Ya-1

In this study, a non-synonymous SNP at position 1931 in the *gB* genes from the field isolates, except for Farm Ya-5, was cytosine, was similar to what is seen in most field strains, whereas the one from most vaccine strains was coded for thymine (Table 3). This point mutation led to an isoleucine-to-threonine substitution at position 644 (I644T) in the *gB* protein of field strains. Similarly, some SNPs were observed in the *gJ* and *gG* genes of ILTV. The sequences of the *gJ* gene fragments from the Myanmar isolates, except for Farm Ya-5, were identical to those from a China/K317 vaccine-derived strain and a Korean field strain, whereas the sequences of the *gG* gene fragments from the Myanmar isolates were unique (Table 3). Moreover, a point mutation in position 102 in the *gG* gene led to a non-synonymous amino acid substitution (Glutamic acid-to-Aspartic acid substitution at position 34, E34D). Five distinct haplotypes were defined according to the specific changes in select nucleotide positions of the *gJ* gene [1]. Sequence analysis in the present study showed that haplotype 2 was the predominant type (Supplemental Table 2).

### Phylogenetic analysis of the ICP4, gB, gG and gJ genes

**Fig. 1:**
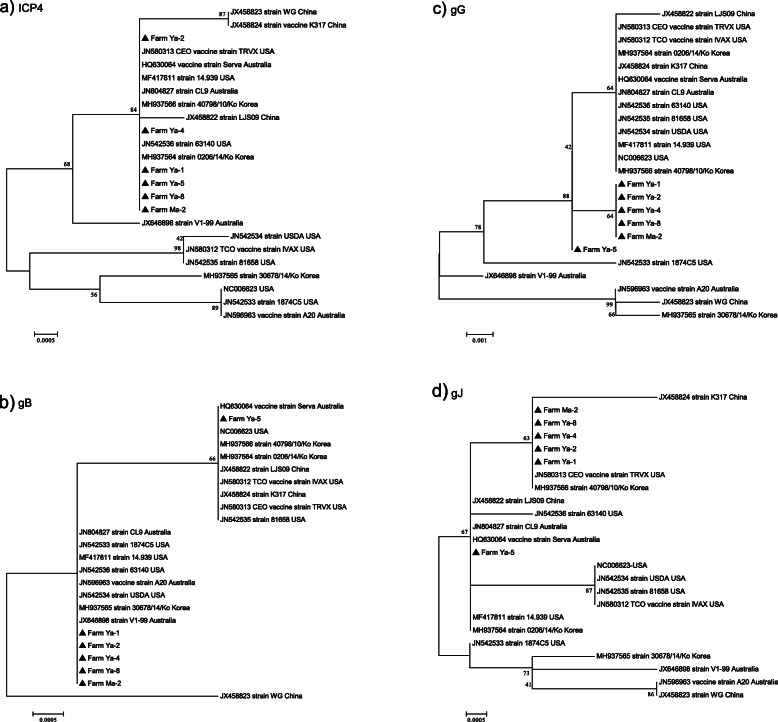
The phylogenetic trees based on the alignment of the Myanmar isolates and reference strains from four different gene fragments: (**a**) *ICP4*, (**b**) *gB*, (**c**) *gG*, and (**d**) *gJ*. The trees were generated using the neighbor-joining method coupled with the Kimura 2-parameter model and a bootstrap analysis of 1000 replicates

Phylogenetic analysis based on the ICP4 and gB genes showed that five out of six isolates obtained in this study clustered together and were closely related to reference strains, including from Asian countries (Fig. 1a and b). In contrast, the phylogenetic tree constructed using the *gG* and *gJ* genes showed that the five isolates in Myanmar formed into a distinct cluster, separate from other reference strains deposited in the GenBank database (Fig. 1c and d).

## Discussion

Although ILTV causes less mortality than the highly pathogenic avian influenza virus and Newcastle disease, its impact on avian productivity has caused significant economic losses to the poultry industry worldwide [22]. However, no scientific data on ILTV surveillance in poultry farms in Myanmar has been available until now. In this study, we investigated the presence of ILTV in Myanmar among 20 poultry farms in Myanmar using PCR targeting the TK gene and we detected ILTV in six farms that were located in southern Myanmar.

Molecular characterization of ILTV is required to differentiate between field and vaccine strains [21, 23, 24]. *ICP4* is responsible for the regulation of gene expression early in infection [25] and has been proposed as a potential differentiation marker due to differences in this gene in the wild-type and vaccine strains [26]. The sequences from the isolates in Myanmar in the present study had a 12 bp-deletion at positions 259–270 in the *ICP4* gene fragment 1; this deletion is typically not present in the TCO vaccine strains. In addition, the nucleotide sequences of *ICP4* gene fragment 2 in the isolates showed distinct differences from TCO vaccine strain sequences. According to the local veterinarians from Myanmar poultry farms, TCO vaccine strain is used to prevent the incidence of ILT in poultry farms that we visited. Therefore, the isolates detected in the present study appear to be field strains.

Glycoprotein B encoded by *UL27* gene is one of the major proteins in ILTV, playing a fundamental role in virus attachment to target cells and cell entry [27]. According to our data, the point mutation at position 1931 in the *gB* gene was found in most virulent and vaccine strains (including TCO and CEO strains). Gracía et al. also reported that the codon at position 1931 in the *gB* gene from most field strains was coded for cytosine, whereas the one from most vaccine strains was coded for thymine [28]. Therefore, the SNP at position 1931 in the *gB* gene could act as a good differentiation marker for field and vaccine strains [9, 28]. In contrast, the isolate from Farm Ya-5 showed similarity to the vaccine strains as well as a few field isolates.

gJ protein is a major viral antigen and plays an important role during egress of ILTV [29]. Craig et al. [1] compared seven different partial fragments of some ILTV genes (*TK*, *gD*, *gG*, *gB*, *gC*, *gJ*, and *ICP4*). The gJ sequence was the most informative segment to discriminate between field and vaccine strains [1], and the gJ sequence of the isolates in the present study indicated haplotype 2 out of five distinct haplotypes.

Sequencing analysis of the *gG* gene has also been used to characterize ILTV isolates [30]. By comparing the partial sequence of *gG* genes with those of other reference strains, a non-synonymous substitution (Glu-to-Asp) at position 34 was identified in the *gG* gene of field isolates from this study. To our knowledge, no other studies have reported this mutation in the *gG* gene of either field or vaccine strains. Further investigation of ILTV strains circulating in the other regions of Myanmar is therefore necessary. Furthermore, since ILTV gG is a known virulence factor that can bind chemokines with high affinity and inhibit leukocyte chemotaxis [31, 32], the biological significance of this amino acid substitution (Glu 34 Asp) in the *gG* gene requires further investigation to determine whether it impacts on the pathogenicity of ILTV.

In the present study, ILTV was mainly detected in the Yangon farms (southern area of the country). All the Yangon samples were collected in May, which is the wet season in Myanmar. In contrast, the Mandalay and Pyin Oo Lwin samples were collected in February, which is the dry season, and almost all were negative for ILTV. The duration of sunshine in the dry season is longer than during the wet season in Myanmar. Since the ultraviolet rays in sunlight might affect the activity of ILTV, it is possible that ILTV transmission may be limited during the dry season, thus partially explaining why most positive samples were detected from Yangon farms and very few from Mandalay and Pyin Oo Lwin farms. Therefore, future studies should ensure that sampling is conducted during similar seasons to ensure accurate representation of the circulating ILTV strains in Myanmar.

Phylogenetic analyses of the *ICP4* and *gB* genes indicated that the Myanmar ILTV isolates were closely related to ILTV reference strains including Asian strains, especially three Korean field isolates, which most likely originated from the Serva vaccine strain [10]. These results suggest that the ILTV isolates detected in poultry farms in Myanmar might be similar to those circulating in neighboring Asian countries, and they have perhaps been endemic for a certain time given the presence of the unique mutations in the *gG* and *gJ* genes. According to the phylogenetic analysis comparing the *gB* and *gG* gene sequences obtained in this study and those previously published in Genbank, five Myanmar isolates clustered into separate branches belonging to the CEO vaccine and TCO vaccine strains. In contrast, phylogenetic analysis using the *gJ* and *ICP4* gene sequences revealed that these isolates clustered together with CEO vaccine. In a previous study by Oldoni et al. [33], three isolates could only be differentiated from the CEO vaccine by the analysis of glycoprotein M gene. Meanwhile, molecular techniques have identified live-attenuated vaccines as one of the main causes of ILTV outbreaks worldwide [8]. CEO vaccine has been banned in Argentina for more than 10 years due to its associated reversion to virulence [1]. Shehata et al. [34] also isolated three highly pathogenic CEO-like field strains and suggested that CEO vaccine strains could increase in virulence after bird-to-bird passages causing severe outbreaks in susceptible birds. It is more likely that the ILTV isolates circulating in poultry farms in Myanmar originated from CEO-like viruses. However, such a hypothesis requires further periodical surveillance using larger sample sizes and sequence analysis based on additional ILTV genomic regions.

## Conclusions

This study demonstrated the presence of ILTV in poultry farms in Myanmar. Genetic characterization of the ICP4, gB, gG, and gJ genes indicated that these isolates were different from vaccine strains and seemed to be field strains circulating in Myanmar. Phylogenetic analysis revealed that these isolates clustered together in a single branch and were closely related to other reference strains, in particular Asian isolates. These results provide some fundamental data for epidemiological studies monitoring the spread of ILTV in Myanmar.

## Methods

### Sample collection

Sample collection was conducted as previously reported at 20 chicken farms located in three major poultry-farming areas in Myanmar, namely Mandalay, Pyin Oo Lwin, and Yangon [20]. Briefly, oropharyngeal swabs were collected from five farms in Mandalay and five farms in Pyin Oo Lwin in February 2018, and ten farms in Yangon in May 2018. In each farm, swab samples were collected from six, nine, or twelve adult laying hens whose breeds were Rhode Island Red or White Leghorn (Table 1). The laying hens in these 20 farms were immunized with ILTV live vaccine (LT-IVAX strain) at the age of 10 weeks. All samples were transferred to the laboratory at 4 °C within 2 days of swab collection and were then stored at − 80 °C until use.

### DNA extraction and molecular detection of ILTV

Three swab samples were pooled (Table 1) and DNA was extracted using a QIAamp DNA Mini Kit (Qiagen, Hilden, Germany) according to the manufacturer’s instructions. The extracted DNA samples were stored at − 20 °C until use.

The thymidine kinase (*TK*) gene of ILTV was targeted for the detection of ILTV by PCR using previously published primers (Table 4) [35]. The PCR mixture contained 10 pmol of each primer, 1 U of TaKaRa Ex Taq (TaKaRa Bio Inc., Kusatsu, Japan), and 200 μM of each deoxynucleotide (TaKaRa Bio Inc.). The DNA sample obtained from an attenuated ILTV live vaccine (LT-IVAX strain) (Kyoritsu Seiyaku Corporation) was used as a positive control.

**Table 4:** Primers used for amplification of each gene in this study

Target gene	Primer name	Primer sequences (5′ – 3′)	PCR conditions	Expected size (bp)	References
For detection of pathogen					
TK	TK-F	ACG ATG ACT CCG ACT TTC	94 °C 2 min; 35 × (94 °C 30s, 55 °C 30s, 72 °C 50s); 72 °C 10 min	647	Pang et al. [35]
	TK-R	CGT TGG AGG TAG GTG GTA			
For sequence analysis					
gB	gB-F	CAA GGG CGG AAT TTG ATA GA	94 °C 2 min; 35 × (94 °C 30s, 55 °C 30s, 72 °C 50s); 72 °C 10 min	440	This study
	gB-R	AAT GAG GCG ATG CCA GAT GC			
gG	gG-F	TTG TGC GCG TCT GTA TTA GG	94 °C 2 min; 35 × (94 °C 30s, 55 °C 30s, 72 °C 30s); 72 °C 10 min	612	This study
	gG-R	CTC CAT AGG ACC GTC GAG TT			
gJ	gJ-F	GTT AAC GCC TCT CTG GAA CG	94 °C 2 min; 35 × (94 °C 30s, 55 °C 30s, 72 °C 50s); 72 °C 10 min	667	This study
	gJ-R	TCG GGG AAG TAC CTG TAT CG			
ICP4 fragment 1	ICP4a-F	ACT GAT AGC TTT TCG TAC AGC ACG	94 °C 2 min; 35 × (94 °C 30s, 55 °C 30s, 72 °C 50s); 72 °C 10 min	688	Chacon et al. [21]
	ICP4a-R	CAT CGG GAC ATT CTC CAG GTA GCA			
ICP4 fragment 2	ICP4b-F	CGA AAT CGG AAA AGC TTC AG	94 °C 2 min; 35 × (94 °C 30s, 55 °C 30s, 72 °C 50s); 72 °C 10 min	624	This study
	ICP4b-R	CTC CAG CAA CAA CAC ATT GG			

### Genetic characterization of ILTV

DNA samples positive for TK gene were subjected to sequencing analysis of *ICP4*, *gB*, *gG*, and *gJ* genes. For each gene, the regions in which the polymorphisms are typically observed among ILTV strains were partially amplified by PCR (Table 4) [21]. For sequencing, the amplified DNA products were purified using a FastGene gel/PCR extraction kit (NIPPON Genetics Co. Ltd.), and the nucleotide sequences were determined using the GenomeLab™ GeXP Genetic Analysis System (Beckman Coulter, Fullerton, CA, USA). The obtained sequences of the *ICP4, gB, gG, gJ* genes were aligned with reference sequences from GenBank database (Table 5) using MEGA6 software [37] and the phylogenetic trees were generated with the same software using the neighbor-joining method coupled with Kimura 2-parameter model with bootstrap analysis of 1000 replicates [38].

**Table 5:** Reference strains used in this study

Virus strains	Origin	Country	Accession
14.939	Field strain	U.S.	MF417811
1874C5	Field strain	U.S.	JN542533
USDA	Challenge strain	U.S.	JN542534
81,658	Field strain	U.S.	JN542535
63,140	Field strain	U.S.	JN542536
CL9	Field strain	Australia	JN804827
V1–99	Field strain	Australia	JX646898
A20	Vaccine strain	Australia	JN596963
Serva	Vaccine strain	Australia	HQ630064
LJS09	Field strain	China	JX458822
WG	Field strain	China	JX458823
K317	Vaccine strain	China	JX458824
0206/14/Ko	Field strain	Korea	MH937564
30,678/14/Ko	Field strain	Korea	MH937565
40,798/10/Ko	Field strain	Korea	MH937566
TCO-IVAX	Vaccine strain	U.S.	JN580312
CEO-TRVX	Vaccine strain	U.S.	JN580313
Gallid herpesvirus 1	N/A	N/A	NC006623 ^a^

*N/A* Not applicable

^a^The ILTV DNA sequence was assembled from 14 published ILTV sequences [36]

## Supplementary Information

**Additional file 1:** **Table S1.** Nucleotide sequence alignment of Tk gene fragments from the isolates in Myanmar, vaccines strains and other ILTV strains. **Table S2.** Defined haplotype according to gJ amplified sequence.

## Data Availability

The datasets analysed during the current study are available in the the National Center for Biotechnology Information (NCBI) repository, accession numbers: LC592178–213.

## Abbreviations

CEO: Chicken embryo-origin
GaHV-1: *Gallid alphaherpesvirus 1*
ILTV: Infectious laryngotracheitis virus
ILT: Infectious laryngotracheitis
PCR: Polymerase chain reaction
SNP: Single nucleotide polymorphisms
TCO: Tissue culture-origin
TK: Thymidine kinase
