# Supplementary material for: Molecular detection and genetic characterization of infectious laryngotracheitis virus in poultry in Myanmar
Source: BMC Vet Res. 2020 Nov 23;16:453. doi: 10.1186/s12917-020-02666-z (PMC7685572; doi:10.1186/s12917-020-02666-z)
Supplement: Supplementary file 1 — Additional file 1: Table S1. Nucleotide sequence alignment of Tk gene fragments from the isolates in Myanmar, vaccines strains and other ILTV strains. Table S2. Defined haplotype according to gJ amplified sequence. [file 12917_2020_2666_MOESM1_ESM.docx]

**Supplemental Table 1 Nucleotide sequence alignment of Tk gene fragments from the isolates in Myanmar, vaccines strains and other ILTV strains**

| Name of strains | Nucleotide position from ATG^a^ of TK gene | | | | | |
| --- | --- | --- | --- | --- | --- | --- |
|  | 289 | 304 | 441 | 594 | 634 | 755 |
| Farm Ya-1 | C | C | G | A | G | C |
| Farm Ya-2 | －^b^ | － | － | － | － | － |
| Farm Ya-4 | － | － | － | － | － | － |
| Farm Ya-5 | － | － | － | － | － | － |
| Farm Ya-8 | － | － | － | － | － | － |
| Farm Ma-2 | － | － | － | － | － | － |
| MF417811_USA/14.939 | － | － | － | － | － | － |
| JN542533_USA/1874C5 | － | － | － | － | A | － |
| JN542534_USA/USDA | － | － | － | － | － | － |
| JN542535_USA/81658 | － | － | － | － | － | － |
| JN542536_USA/63140 | T | － | － | － | － | － |
| JN804827_Australia/CL9 | － | － | － | C | － | T |
| JX646898_Australia/V1-99 | － | － | A | － | － | － |
| JN596963_Australia/A20 vaccine | － | － | － | C | － | T |
| HQ630064_Australia/Serva vaccine | － | － | － | － | － | － |
| JX458822_China/LJS09 | － | － | － | － | － | － |
| JX458823_China/WG | － | － | － | C | － | T |
| JX458824_China/K317 vaccine | － | － | － | － | － | － |
| MH937564_Korea | － | － | － | － | － | － |
| MH937565_Korea | － | － | － | － | － | － |
| MH937566_Korea | － | － | － | － | － | － |
| JN580312/TCO vaccine-IVAX | － | － | － | － | － | － |
| JN580313/CEO vaccine-TRVX | － | － | － | － | － | － |
| NC006623_USA | － | T | － | － | － | － |

^a^ The sequence of TK gene with Genbank accession number NC_006623 was taken as a reference.

^b^ －Regions where the sequences are identical to those of Farm Ya-1.

**Supplemental Table 2 Defined haplotype according to gJ amplified sequence**

| Name of strains | Nucleotide position from ATG ^a^ of gJ gene | | | | | Haplotype ^b^ |
| --- | --- | --- | --- | --- | --- | --- |
|  | 461 | 484 | 832 | 878 | 894 |  |
| Farm Ya-1 | A | C | A | T | G | 2 |
| Farm Ya-2 | －^c^ | － | － | － | － | 2 |
| Farm Ya-4 | － | － | － | － | － | 2 |
| Farm Ya-5 | － | － | － | － | － | 2 |
| Farm Ya-8 | － | － | － | － | － | 2 |
| Farm Ma-2 | － | － | － | － | － | 2 |
| MF443837/ER07_01 | － | － | G | － | － | 5 |
| MF443847/ER08_07 | － | － | G | － | － | 5 |
| MF443828/ER08_03 | － | － | － | － | A | 4 |
| MF443829/ER08_05 | － | － | － | － | A | 4 |
| MF443826/ER10_01 | T | － | － | － | － | 3 |
| MF443807/BA09_071 | T | － | － | － | － | 3 |
| MF443798/RN09_109 | － | － | － | － | － | 2 |
| MF443822/ER06_01 | － | － | － | － | － | 2 |
| JN580312/TCO vaccine-IVAX | － | T | － | C | － | 1 |
| JN580313/CEO vaccine-TRVX | － | － | － | － | － | 2 |
| NC006623_USA | － | T | － | C | － | 1 |

^a^ The sequence of gJ gene with Genbank accession number NC_006623 was taken as a reference.

^b^ The five haplotypes were defined based on the previous report [1].

^c^ －Regions where the sequences are identical to those of Farm Ya-1.
